# Retraction: Downregulation of X-linked inhibitor of apoptosis protein by ‘7-Benzylidenenaltrexone maleate’ sensitizes pancreatic cancer cells to TRAIL-induced apoptosis

**DOI:** 10.18632/oncotarget.28813

**Published:** 2025-12-31

**Authors:** So Young Kim, Sojung Park, SeonA Yoo, Jin Kyung Rho, Eun Sung Jun, Suhwan Chang, Kyung Kon Kim, Song Cheol Kim, Inki Kim

**Affiliations:** ^1^ASAN Institute for Life Sciences, ASAN Medical Center, Seoul, 05505, South Korea; ^2^Department of Convergence Medicine, University of Ulsan College of Medicine, Seoul, 05505, South Korea; ^3^Department of Biomedical Sciences, University of Ulsan College of Medicine, Seoul, 05505, South Korea; ^4^Division of HBP Surgery, Department of Surgery, University of Ulsan College of Medicine, Asan Medical Center, Seoul, 05505, South Korea

**This article has been retracted:** The journal initiated an investigation into this paper after the authors requested a correction for six Western blot images (Figures 2A, 3A, 5A–5C, and Supplementary Figure 3B) without providing a detailed explanation of the errors. Our investigation found two instances of image duplication:

1. The “α-Tubulin” blot in Figure 2A was rescaled and reused as the “α-Tubulin” blot in Figure 5C.
2. The “Survivin” blot in Figure 3A is a reuse of the “p-CDK9 (T186)” blot previously published in Figure 5C of an earlier article from the same research group [1].

The authors were contacted for comment but did not respond. Due to the number and nature of these issues, the editorial decision was made to retract this paper.

Original article: Oncotarget. 2017; 8:61057–61071. 61057-61071. https://doi.org/10.18632/oncotarget.17841
